# Supplementary material for: Gut bacteria are rarely shared by co-hospitalized premature infants, regardless of necrotizing enterocolitis development
Source: eLife. 2015 Mar 3;4:e05477. doi: 10.7554/eLife.05477 (PMC4384745; doi:10.7554/eLife.05477)
Supplement: Supplementary file 5. — Analysis of deeply sampled Enterococcus faecalis populations to detect sequencing reads with SNPs consistent with their derivation from populations present in other infants. Less deeply sampled populations typically had no SNPs consistent with derivation from a population present in another infant. DOI: http://dx.doi.org/10.7554/eLife.05477.024 [file elife05477s005.docx]

**Table S5:** Analysis of deeply sampled *E. facealis* populations to detect sequencing reads with SNPs consistent with their derivation from populations present in other infants.

**
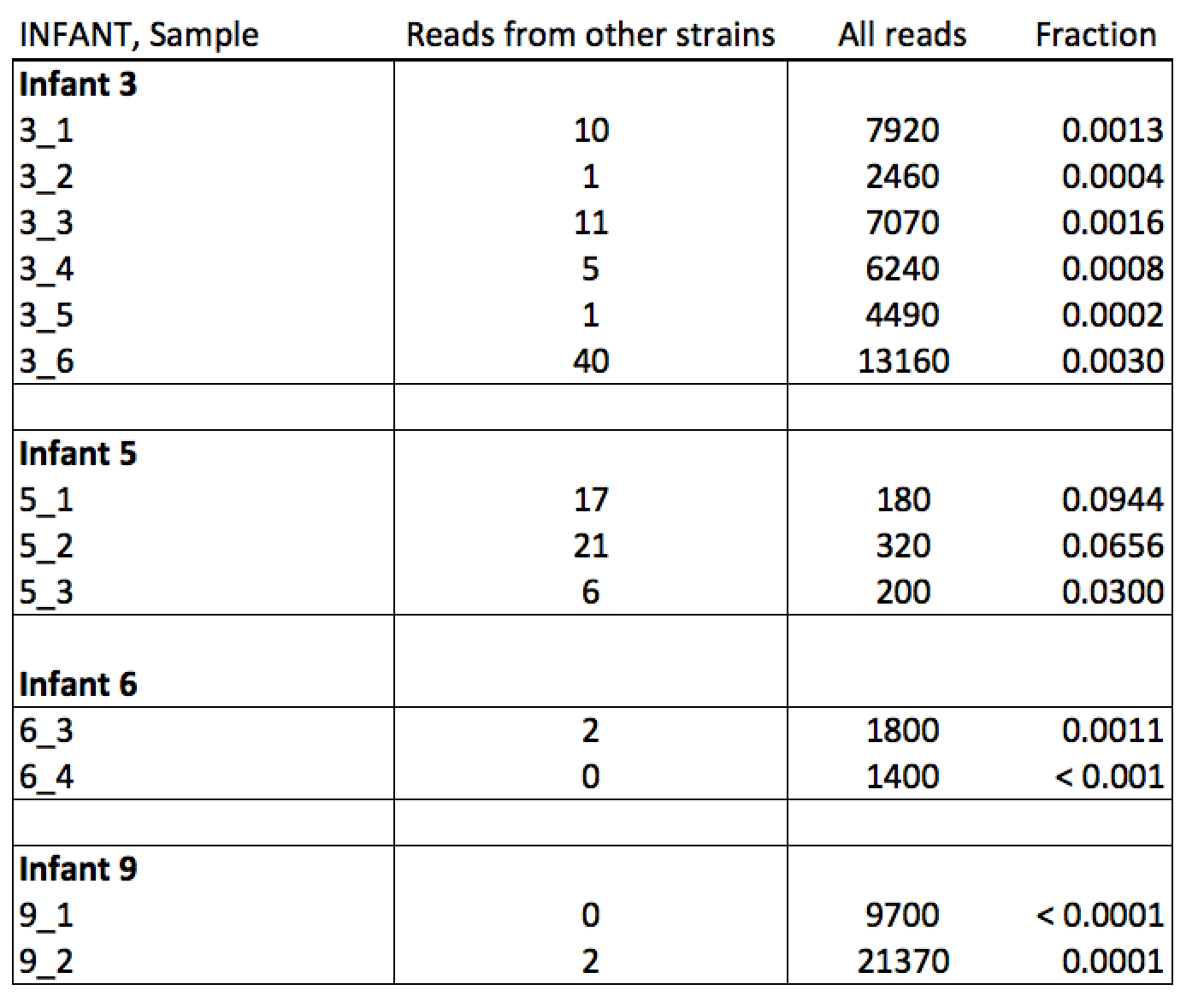
**
