# Supplementary material for: Gut bacteria are rarely shared by co-hospitalized premature infants, regardless of necrotizing enterocolitis development
Source: eLife. 2015 Mar 3;4:e05477. doi: 10.7554/eLife.05477 (PMC4384745; doi:10.7554/eLife.05477)
Supplement: Supplementary file 6. — In no case does Enterococcus faecalis have a spacer that can silence a phage present in the same community. Green text highlights cases where mutations likely prevent silencing. DOI: http://dx.doi.org/10.7554/eLife.05477.025 [file elife05477s006.docx]

**Table S6** In no case does *E. faecalis* have a spacer that can silence a phage present in the same community.

| Spacer | Phage or Prophage | Comments | Mismatches |
| --- | --- | --- | --- |
| **Infant 5, sample 2,3,4**  (NECEvent2014_5_2_scaffold_206)  (NECEvent2014_5_3_scaffold_171)  (NECEvent2014_5_4_scaffold_877)  **Infant 3, samples 1 - 6**  (NECEvent2014_3_1_scaffold_28)  (NECEvent2014_3_2_scaffold_21)  (NECEvent2014_3_3_scaffold_39)  (NECEvent2014_3_4_scaffold_56)  (NECEvent2014_3_5_scaffold_38)  (NECEvent2014_3_6_scaffold_62) | **Infant 3, sample 6**  *E. faecalis* phage   - (NECEvent2014_3_6_scaffold_139) |  | 3 |
| **Infant 5, sample 2-4**  (NECEvent2014_5_2_scaffold_206)  (NECEvent2014_5_3_scaffold_171)  (NECEvent2014_5_4_scaffold_877)  **Infant 3, samples 1- 6**  (NECEvent2014_3_1_scaffold_28)  (NECEvent2014_3_2_scaffold_21)  (NECEvent2014_3_3_scaffold_39)  (NECEvent2014_3_4_scaffold_56)  (NECEvent2014_3_5_scaffold_38)  (NECEvent2014_3_6_scaffold_62) | **Infant 5, sample 7**  *E. faecalis* phage   - (NECEvent2014_5_7_scaffold_888)   **Infant 5, sample 8**  *E. faecalis* phage   - (NECEvent2014_5_8_scaffold_404) |  | 0  0 |
| **Infant 9, sample 1,2**  (NECEvent2014_9_1_scaffold_0)  (NECEvent2014_9_2_scaffold_0) | **Infant 9, samples 1 and 2**  *E. faecalis* prophage   - (NECEvent2014_9_1_scaffold_39)   (NECEvent2014_9_2_scaffold_36)  **Infant 5, samples 7 and 8**  *E. faecalis* prophage  (NECEvent2014_5_7_scaffold_244)  (NECEvent2014_5_8_scaffold_129) | The spacer is similar to a spacer from **Infant 8, sample 7** (NECEvent2014_8_7_scaffold_124), see below. | 2  1 |
| **Infant 8, sample 1-5,7**  (NECEvent2014_8_1_scaffold_184)  (NECEvent2014_8_2_scaffold_21)  (NECEvent2014_8_3_scaffold_145)  (NECEvent2014_8_4_scaffold_126)  (NECEvent2014_8_5_scaffold_56)  (NECEvent2014_8_7_scaffold_124) | **Infant 5, sample 8**  *E. faecalis* prophage  (NECEvent2014_5_8_scaffold_129)  **Infant 5, sample 7**  *E. faecalis* prophage  (NECEvent2014_5_7_scaffold_244) | Related (but not identical) to a prophage in **Infant 9, samples 1 and 2** (see above) and in three samples from **Infant 2**. | 0  0 |
